# Decorin Mimic Inhibits Vascular Smooth Muscle Proliferation and Migration

**DOI:** 10.1371/journal.pone.0082456

**Published:** 2013-11-22

**Authors:** Rebecca A. Scott, John E. Paderi, Michael Sturek, Alyssa Panitch

**Affiliations:** 1 Weldon School of Biomedical Engineering, Purdue University, West Lafayette, Indiana, United States of America; 2 Department of Cellular and Integrative Physiology, Indiana University School of Medicine, Indianapolis, Indiana, United States of America; IDI, Istituto Dermopatico dell'Immacolata, Italy

## Abstract

Over the past 10 years, the number of percutaneous coronary intervention procedures performed in the United States increased by 33%; however, restenosis, which inhibits complete functional recovery of the vessel wall, complicates this procedure. A wide range of anti-restenotic therapeutics have been developed, although many elicit non-specific effects that compromise vessel healing. Drawing inspiration from biologically-relevant molecules, our lab developed a mimic of the natural proteoglycan decorin, termed DS-SILY, which can mask exposed collagen and thereby effectively decrease platelet activation, thus contributing to suppression of vascular intimal hyperplasia. Here, we characterize the effects of DS-SILY on both proliferative and quiescent human SMCs to evaluate the potential impact of DS-SILY-SMC interaction on restenosis, and further characterize *in vivo* platelet interactions. DS-SILY decreased proliferative SMC proliferation and pro-inflammatory cytokine secretion *in vitro* in a concentration dependent manner as compared to untreated controls. The addition of DS-SILY to *in vitro* SMC cultures decreased SMC migration and protein synthesis by 95% and 37%, respectively. Furthermore, DS-SILY decreased platelet activation, as well as reduced neointimal hyperplasia by 60%, *in vivo* using Ossabaw swine. These results indicate that DS-SILY demonstrates multiple biological activities that may all synergistically contribute to an improved treatment paradigm for balloon angioplasty.

## Introduction

Percutaneous coronary intervention (PCI) is an invasive cardiovascular procedure performed to mechanically widen narrowed coronary vessels using either balloon angioplasty or intracoronary stenting. While the number of PCI procedures performed in the United States has increased by 33% in the past 10 years[1-3], thrombosis, neointimal hyperplasia, and restenosis remain complications of this procedure. The occurrence of these detrimental consequences following PCI is attributed to trauma during the procedure, which triggers an array of mechanical and biological processes implicated in the healing process. 

PCI often damages the endothelial cells (EC) from the vessel wall, exposing the underlying collagenous layer and providing inherent targets for platelet activation[4,5]. In addition, the injured endothelium produces a plethora of pro-inflammatory and mitogenic substances, all of which leads to the recruitment of inflammatory cells to the site of injury, via surface expression of adhesion molecules, and ultimately the production of a range of pro-inflammatory factors[6-8]. Vascular smooth muscle cells (SMC) remain targets for many of the pro-inflammatory factors released from activated inflammatory cells and platelets[9,10]. As such, activated SMCs are stimulated to proliferate and migrate into the neointimal layer of the vessel wall, as well as synthesize new extracellular matrix[10-14]. Moreover, stimulated SMCs actively participate in the inflammatory cascade, producing and secreting a range of factors, including interleukin-1β (IL-1β), interleukin-6 (IL-6), and tumor necrosis factor-α (TNF-α)[9,10].

Advances within the pharmaceutical and scientific communities have enabled medical researchers to develop a wide range of anti-restenotic therapeutics. The overall goal of the developed therapies is to target the key processes involved in the healing response leading to restenosis, including platelet activation, inflammation, SMC proliferation and migration, and extracellular matrix synthesis[15,16]. An overwhelming number of these therapeutics, including both paclitaxel and sirolimus analogs that are widely used on drug-eluting stents, have been shown to effectively target and inhibit SMC proliferation[17-19]. However, these compounds are non-selective cytotoxins and as such, the effects of the compounds are not limited purely to SMCs, but also to ECs[17]. The non-specific effects of these therapeutics inhibit EC proliferation and migration[18,19], which have been associated with incomplete vessel healing *in vivo*[20].

In an effort to create alternative means of treating restenosis, several research groups have investigated the use of biologically-relevant molecules for anti-restenotic treatment, turning to natural molecules prevalent in the native vessel wall. One such molecule is decorin, a small proteoglycan consisting of a single glycosaminoglycan (GAG) side chain linked to a core protein, which accounts for approximately 22% of the proteoglycans found in the vessel wall[21,22]. Decorin plays a significant role in the regulation of cell migration[23-26], proliferation[23,27], and attachment[25,26,28]. The addition of decorin in both *in vitro* and *in vivo* studies examining restenotic treatment has resulted in decreased SMC proliferation, SMC migration, and collagen synthesis, but exhibited no effect on EC proliferation and migration[23,25]. Furthermore, 10 weeks after PCI, inflammatory cells were almost completely absent from vessels treated with decorin[23]. However, as decorin is relatively expensive and can be readily degraded due to its natural presence in the body, some limitations exist for use of this molecule in clinical applications.

Our lab synthesized a mimic of the proteoglycan decorin[29,30]. This mimic, termed DS-SILY, consists of type-I collagen-binding peptides bound to a dermatan sulfate (DS) backbone. This anti-thrombotic biomolecule has been shown to specifically bind to type-I collagen, serving as a barrier to platelet adhesion and activation *in vitro*[30]. Furthermore, DS-SILY stimulated EC migration on collagen-coated substrates and delivery of this decorin mimic from porous balloons in porcine vessels resulted in significantly decreased arterial vasospasm compared to vessels treated with saline solution[30]. 

As treatment of SMCs with decorin has previously demonstrated inhibition of SMC proliferation and migration, we further characterized the effects of our decorin mimic, DS-SILY, on the anti-restenotic properties of SMCs. We demonstrate here the use of this antithrombotic biomolecule to control SMC migration, protein synthesis, cytokine excretion, and vascular injury marker production of both proliferative and quiescent SMCs *in vitro*. Furthermore, we examine the effects of this molecule on platelet adhesion and activation *in vivo*, as well as on intimal hyperplasia in Ossabaw swine.

## Materials and Methods

### DS-SILY_20_ Synthesis

The decorin mimic (DS-SILY) was synthesized as previously described[30]. Briefly, carboxyl groups present on the backbone of dermatan sulfate (DS, MW 46,275 Da, Celsus Laboratories) were oxidized via standard periodate oxidation to form aldehyde moieties. Oxidized DS was then covalently coupled to the heterobifunctional crosslinker *N*-[b-maleimidopropionic acid] hydrazide, trifluoroacetic acid salt (BMPH, Thermo Fisher Scientific) in phosphate buffered saline (PBS). The collagen-binding peptide sequence RRANAALKAGELYKSILYGC (noted as SILY, Genscript), derived from the platelet receptor to type I collagen, was conjugated to the DS-BMPH compound; specifically, the thiol group on the cysteine amino acid reacted with the maleimide group of BMPH to form a thioether bond. Purifications were performed at each step by size exclusion chromatography and the number of attached peptides was determined by the consumption of BMPH in the second reaction step. The final product DS-SILY_n_, where *n* indicates the number of attached SILY peptides, was purified in ultrapure H_2_O, lyophilized and stored at -20°C until use.

### Cell Culture

Human coronary artery smooth muscle cells (SMC, Invitrogen) were cultured in Media 231 (M231, Invitrogen), supplemented with 4.9% fetal bovine serum (FBS, Invitrogen), 2 ng/mL basic fibroblast growth factor (Invitrogen), 0.5% epidermal growth factor (Invitrogen), 5 ng/mL heparin (Invitrogen), 5 μg/mL insulin (Invitrogen), and 0.2 μg/mL bovine serum albumin (BSA, Invitrogen). Unless otherwise noted, cells were initially seeded at 5x10^4^ cells/cm^2^ in Ibidi angiogenesis μ-slide (Ibidi) and allowed to proliferate for 24 hrs to allow the formation of multilayered cell constructs. Media was removed and cultures were treated either with proliferative media, as described above, or contractile media to induce a quiescent phenotype, for 24 hrs. Previously, we have demonstrated that the addition of contractile media, consisting of M231 supplemented with 1% FBS and 30 μg/mL heparin, induced SMCs to transition from a proliferative state to a more differentiated, contractile state due to low serum and introduction of heparin[31]. Treatments were applied to the SMC cultures using co-culture media, which was composed of Media 200 (M200, Invitrogen) with 2% FBS, 1 μg/mL hydrocortisone (Invitrogen), 30 ng/mL heparin, and 0.2 μg/mL BSA. Cells were used between passage numbers 3 and 8 for all assays and maintained at 37°C with 5% CO_2_.

### SMC Metabolic Activity and Proliferation

Cells were incubated in the presence of 0, 0.01, 0.1, 1, or 10 μM DS-SILY_20_ for 24 hrs. The metabolic activity of the cells was determined using the CellTiter 96 AQ_ueous_ One Solution Cell Proliferation Assay (Promega). Briefly, media was mixed with 3-(4,5-dimethylthiazol-2-yl)-5-(3-carboxymethoxyphenyl)-2-(4-sulfophenyl)-2H-tetrazolium, inner salt (MTS) and cultures were re-incubated for 2 hrs at 37°C with 5% CO_2_. The media containing MTS was then transferred into a 96-well plate and absorbance at 490 nm was measured. 

The effect of DS-SILY_20_ on cell proliferation was assessed by determining the number of cells per volume after treatment. Cultures were fixed with 4% formaldehyde and nuclei were stained using SYTOX green (Invitrogen). Cells were visualized using an Olympus FV1000 confocal microscope with 60x objective. Scans were completed with a xy area of 512 μm^2^ and one stack, 14 μm (1 μm per step) in the z-direction, was taken at three separate locations in each culture. Cell nuclei were counted, such that cell proliferation was assessed by determining the number of SMC nuclei per volume.

### Migration

SMC migration was examined via a modified Boyden chamber, using a polycarbonate filter (8.0 μm pore size, Corning) to divide the upper and lower chambers. The lower chamber of each well was filled with serum-free M200 containing 1% BSA. SMCs were trypsinized and resuspended in serum-free M200 containing 1% BSA with or without varying concentrations of DS-SILY_20_. Cells (5x10^4^ cells/cm^2^) were added to the upper portion of the transwell chamber and incubated for 5 hrs at 37°C. Following incubation, cells were fixed in 4% formaldehyde and nuclei stained with Hoechst 33342. Transwells were then mounted on glass slides and migratory SMCs visible on the lower side of the filters were counted by light microscopy using 10x magnification. 

### Protein Synthesis

Cells were incubated in the presence of 0, 0.01, 0.1, 1, or 10 μM DS-SILY_20_ for 24 hrs. Cells were washed twice with ice cold PBS, scraped in lysis buffer (9 M urea, 4% CHAPS, and phosphatase inhibitor cocktail-1 (Sigma) in Millipore water), and frozen overnight at -80°C. Lysates were processed for 3 hrs in a Disruptor Genie (Scientific Industries) at 4°C and centrifuged for 20 min at 18,000×g to remove membrane components. A BCA assay protein kit (Pierce) was used to quantify total protein. 

To determine the effects of DS-SILY_20_ on protein synthesis in both proliferative and differentiated SMC cultures, click chemistry was utilized to fluorescently label newly synthesized proteins (Figure S1)[32]. After washing the cells once with PBS, cells were incubated at 37°C for 60 min with serum-free media to deplete methionine reserves. Cultures were then supplemented with 1 μM L-azidohomoalanine (AHA, Invitrogen) in serum-free media for 4 hrs. Cells were then rinsed with PBS to remove any excess AHA and incubated with co-culture media overnight to allow protein production. Cultures were fixed with 4% formaldehyde, permeabilized with 0.25% Triton X-100 in PBS, and blocked with 1% BSA in PBS. To detect the newly synthesized proteins containing AHA, alkyne-labeled Alexa Fluor 594 (AF-594, Invitrogen) was selectively bound via copper-catalyzed azide-alkyne ligation. Cell nuclei were stained using SYTOX green.

Proteins were visualized using an Olympus FV1000 confocal microscope with 60x objective. Scans were completed with a xy area of 512 μm^2^ and one stack, 14 μm (1 μm per step) in the z-direction, was taken at three separate locations in each culture. Each stack was taken at the same exposure settings to ensure similar darkness values; cultures lacking AHA-treatment were utilized as controls. ImageJ was used to determine the average fluorescent intensity of each stack based on AF-594 fluorescence. Average fluorescent intensity of each stack was then normalized to the number of cells in the 3D image.

### Effect of DS-SILY_20_ on SMC Cytokine Production

Cells were incubated in the presence of 0, 0.01, 0.1, 1, or 10 μM DS-SILY_20_ for 24 hrs. Media was removed from the cultures and a Pro-Inflammatory I kit (Meso Scale Discovery) was used to analyze cytokine production of SMCs according to manufacturer’s instructions. Briefly, plates were warmed to room temperature and incubated with 25 μL of samples and standards for 2 hours at room temperature with vigorous shaking. The detection antibody was then added to the plate and incubated for 2 hours at room temperature with vigorous shaking. After washing three times with PBS with 0.05% Tween-20, 2X read buffer was added to the plate and imaged using a Sector Imager 2400A (Meso Scale Discovery). The pro-inflammatory markers interferon-γ (IFN-γ), interleukin-1β (IL-1β), interleukin-6 (IL-6), and tumor necrosis factor-α (TNF-α) were examined in this study. Data were analyzed using the MSD Discovery Workbench Software.

### Effect of DS-SILY_20_ on SMC Thrombomodulin Production

Following treatment with 0, 0.01, 0.1, 1, or 10 μM DS-SILY_20_ for 24 hrs, cells were washed twice with ice cold PBS, scraped in lysis buffer (9 M urea, 4% CHAPS, and phosphatase inhibitor cocktail-1 in Millipore water), and frozen overnight at -80 °C. Lysates were processed for 3 hrs in a Disruptor Genie (Scientific Industries) at 4°C and centrifuged for 20 min at 18,000×g to remove membrane components. A BCA assay protein kit (Pierce) was used to quantify total protein. A Vascular Injury Marker I kit (Meso Scale Discovery) was used to analyze thrombomodulin production of SMCs according to manufacturer’s instructions. Briefly, plates were warmed to room temperature and incubated with 10 μL of samples and standards for 2 hrs at room temperature with vigorous shaking. Following gentle rinsing of the wells, the detection antibody was then added and incubated for 1 hr at room temperature with vigorous shaking. After washing three times with PBS with 0.05% Tween-20, 2X Read buffer was added to the plate and imaged using a Sector Imager 2400A. Data were analyzed using the MSD Discovery Workbench Software.

### Effect of DS-SILY_20_ on Platelet Deposition and Hyperplasia In Vivo

An Ossabaw pig angioplasty model was used to assess platelet deposition (acute) and neointimal hyperplasia (1-month recovery) after DS-SILY_20_ treatment. Ossabaw pigs under-went angioplasty procedures following approved protocols at the Indiana University School of Medicine similar to previous methods described in detail[33-35]. Each animal received pre-anesthesia with intramuscular injections of xylazine (2.2 mg/kg) and telazol (6.6 mg/kg). Following intubation, isoflurane (2-4%, with oxygen) was administered to maintain stable systemic hemodynamics and a stable level of anesthesia. Under sterile conditions, the right carotid artery was exposed with surgical cut-down technique and an 8F vascular introducer sheath was inserted into the carotid artery followed by administration of heparin (200 Units/kg). An 8F Amplatz left, or other appropriate guiding catheter (Cordis Corporation), was inserted through the sheath and advanced near the target site.

Porous angioplasty balloons (Cook Medical, Bloomington, IN) were employed for delivery of soluble DS-SILY_20_ to the target arteries in the peripheral vasculature of the pigs. Renal, femoral, and iliac arteries were denuded by balloon expansion and movement in the artery, followed by delivery of the therapeutic through the porous balloon. Treatment consisted of 2 mL of 10 μM DS-SILY_20_ dissolved in saline; saline alone was delivered as a sham control. In some cases, bare-metal stents were placed such that they partially overlapped the 20 mm treated artery segment. Stent placement was used as a marker for harvesting treated arteries, and to assess the healing response with stent placement. For each treatment group, 3 animals were utilized.

Arteries for assessing the acute response of balloon injury were harvested within hours of angioplasty. The artery segments were rinsed with saline and fixed in 10% formalin overnight, followed by ethanol dehydration. Arteries were then critical point dried, sputter coated with platinum, and visualized by scanning electron microscopy (SEM). 

Neointimal hyperplasia was assessed by histology after a 1-month recovery. In arteries where stents partially overlapped the treated segment, a gap of several millimeters between the stent and stent-free segment was removed from analysis to avoid stent edge effects. Neointimal hyperplasia was analyzed by measuring the distance from either the internal elastic lamina or a stent post to the lumen in stent-free or stented artery segments, respectively. In two cases, stents were crushed due to placement near a joint, and were excluded from analysis.

### Statistical Analysis

Results are expressed as means ± standard error. Statistical analysis was performed using SAS software (SAS Institute). All results were analyzed using ANOVA with Tukey HSD post-hoc test. The threshold for statistical significance was set at *p*<0.05.

## Results

### SMC Metabolic Activity

To better understand the effects of DS-SILY on SMC behavior, SMCs cultures eliciting either a proliferative or quiescent phenotype were utilized. Quiescent SMC cultures were generated *in vitro* by reducing serum concentrations and adding heparin to SMC media, as previously demonstrated[31]. Using the CellTiter 96 AQ_ueous_ One Solution Cell Proliferation Assay, no change in SMC metabolic activity was exhibited with the addition of any concentration of DS-SILY_20_ (data not shown). This general trend was demonstrated by both the proliferative and quiescent cultures, as compared to controls.

### Proliferation

**Figure 1:**
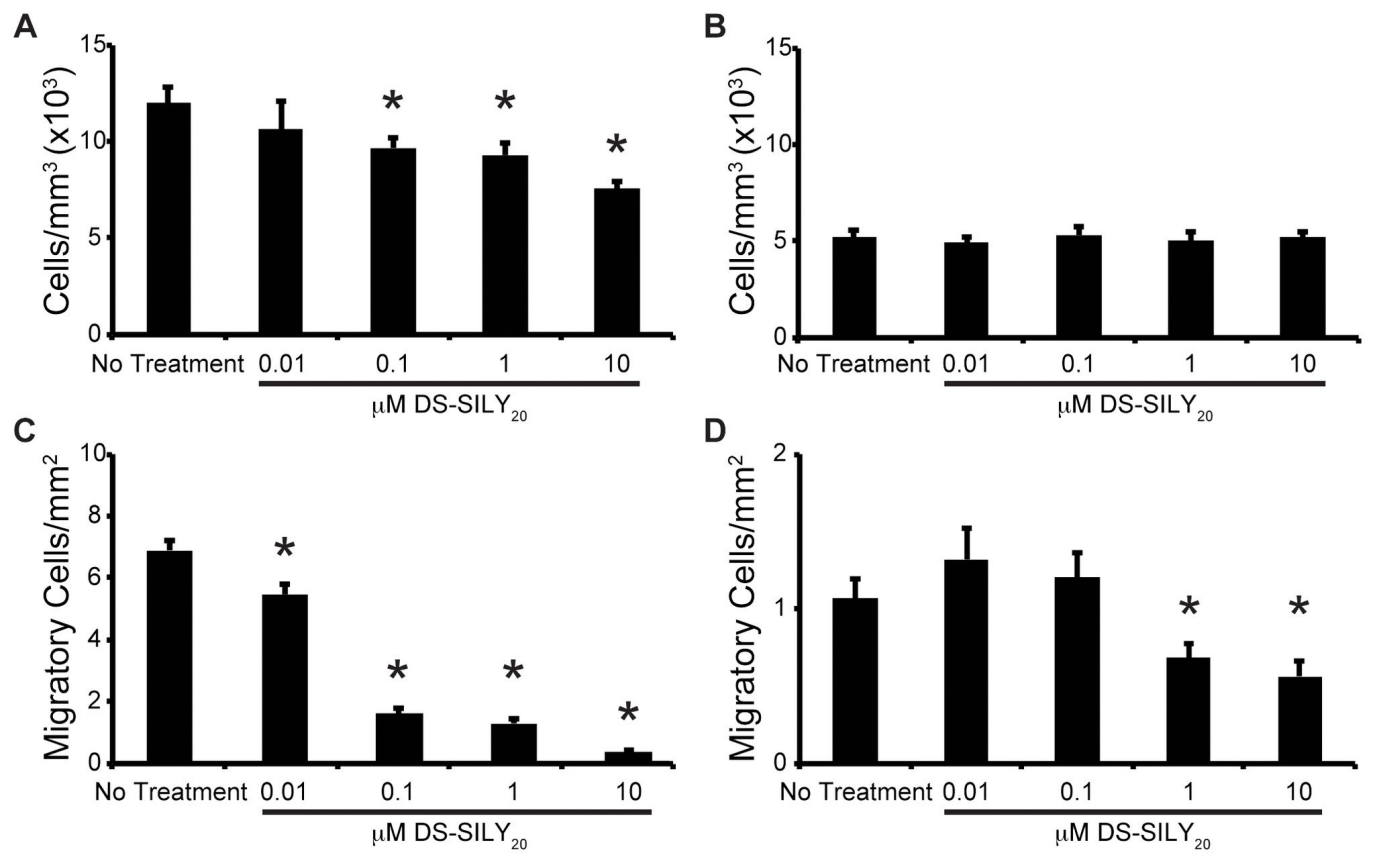
Proliferation and Migration. Proliferation (A, B) and migration (C, D) of proliferative (A, C) and quiescent (B, D) SMCs in response to treatment with DS-SILY_20_. Proliferation and migration of proliferative SMCs significantly decreased with increased concentrations of DS-SILY_20_. Proliferation in quiescent SMC cultures was not altered with addition of DS-SILY_20_ at any concentration; however, quiescent SMC migration decreased with the addition of 1 and 10 μM DS-SILY_20_. * represents significance from control non-treated cells. (N>6).

Proliferation of SMCs was assessed by determining the number of SMC nuclei per cell culture volume following the culture period. Significantly increased numbers of SMC cells were found in proliferative cultures compared to quiescent cultures (Figure 1A & B). For proliferative SMC cultures, the addition of DS-SILY_20_ resulted in decreased proliferation compared to controls, where a significant reduction of SMC proliferation occurred with treatments of 0.1, 1, and 10 μM DS-SILY_20_. While DS-SILY_20_ elicited a response from proliferative cultures, no change in proliferation was exhibited in quiescent SMC cultures.

### Migration

SMC migration was examined using a modified Boyden chamber[36]. Significantly increased migration was exhibited by SMCs in a proliferative phenotype compared to SMCs in a contractile phenotype (Figure 1C & D). The effect of DS-SILY_20_ on SMC migration was examined; a general trend was observed such that as the amount of DS-SILY_20_ increased, SMC migration decreased in both proliferative and quiescent cultures. For proliferative SMCs, a significant decrease in migration was observed for all concentrations of DS-SILY_20_ tested, ultimately culminating with a ~95% decrease in migratory cells in cultures treated 10 μM DS-SILY_20_ compared to controls. The addition of 0.1 and 1 μM DS-SILY_20_ to proliferative cultures decreased migration to a level similar to that of quiescent cells; while treatment of proliferative SMCs with 10 μM DS-SILY_20_ resulted in significantly decreased migration compared to quiescent controls. A significant decrease in the number of migratory cells was only observed in quiescent cultures treated with 1 or 10 μM DS-SILY_20_. 

### Protein synthesis

**Figure 2:**
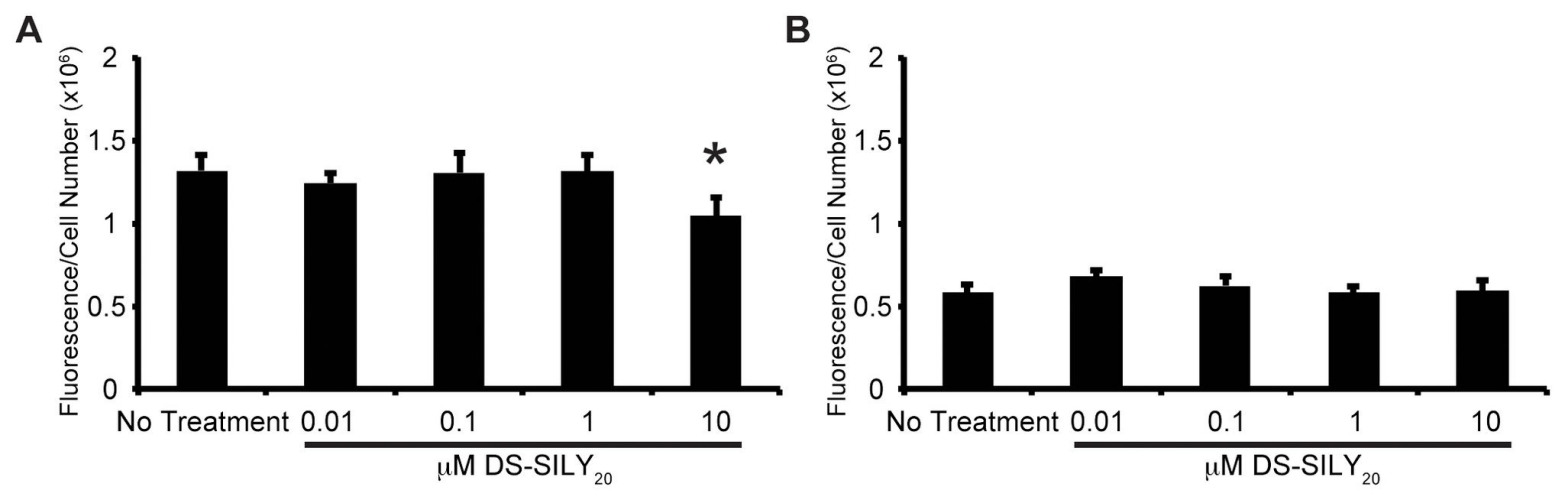
Protein Synthesis. Protein expression of (A) proliferative and (B) quiescent SMCs in response to treatment with DS-SILY_20_. A significant decrease in protein expression was observed in proliferative SMC cultures treated with 10 μM DS-SILY_20_, while no changes in protein synthesis was exhibited in proliferative SMC cultures treated with lower concentrations of DS-SILY_20_ or quiescent cultures with the addition of any concentration DS-SILY_20_. * represents significance from control non-treated cells. (N>6).

Total protein in cultures was assessed via microBCA assay compared to the number of cells per culture, as assessed in proliferation experiments. In proliferative cultures, SMCs produced 3.5 ± 0.3 ng of protein per cell, while only 2.2 ± 0.4 ng of protein per cell was produced by quiescent SMCs. *De novo* protein synthesis following DS-SILY_20_ treatment, and doping with AHA, was analyzed by detecting the presence of the incorporated AHA within proteins via click chemistry reaction, which attached a fluorescent tag directly to the unnatural amino acid. By quantification of fluorescent intensity, proliferative SMC cultures were found to synthesize approximately 65% more protein compared to quiescent cultures (Figure 2). In proliferative SMC cultures, a significant decrease in protein expression was observed in cultures treated with 10 μM DS-SILY_20_, where approximately 37% less protein was synthesized compared to control proliferative cultures. Lower concentrations of DS-SILY_20_ did not elicit a change in protein synthesis in proliferative cultures. Likewise, protein synthesis in quiescent cultures was not altered with the addition of any concentration DS-SILY_20_. 

### Cytokine Expression

**Figure 3:**
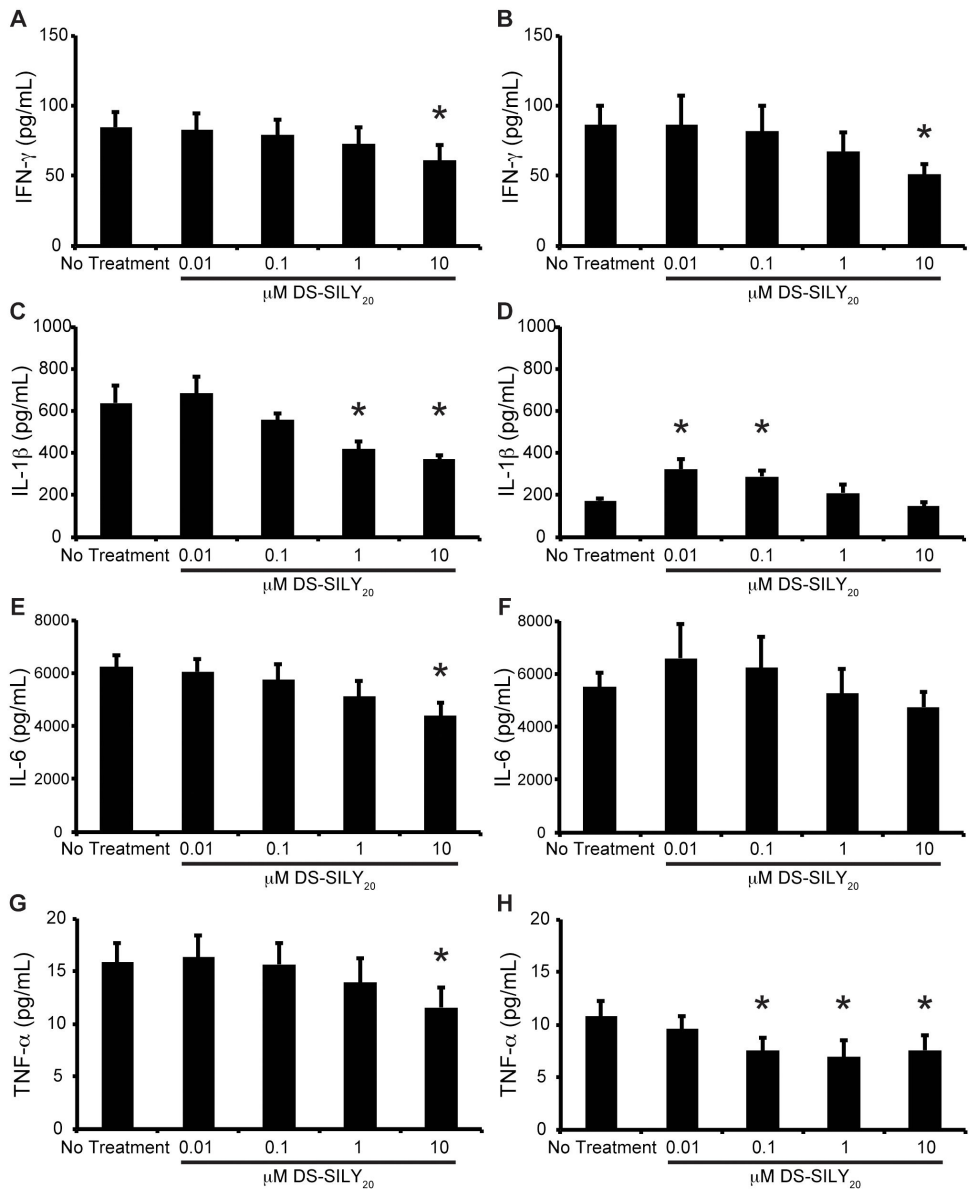
Cytokine Production. Cytokine produced (A, C, E, G) proliferative and (B, D, F, H) quiescent SMCs in response to DS-SILY_20_. The amount of (A, B) IFN-γ, (C, D) IL-1β, (E, F) IL-6, and (G, H) TNF-α produced by cultured SMCs was measured 24 hrs post-treatment. For proliferative SMC cultures, a general trend was observed such that as the concentration of DS-SILY_20_ increased, cytokine production decreased. Significant reductions in IFN-γand TNF-α were also observed in quiescent SMC cultures with the addition of DS-SILY_20_. * represents significance from control non-treated cells. (Nγ6).

Expression of IFN-γ, IL-1β, IL-6, and TNF-α from SMC cultures was examined via MSD Sector Imager. Examination of control, non-treated cultures revealed that proliferative SMCs exhibited increased levels of IL-1β and TNF-α compared to quiescent cultures; however, the two cultures produced similar levels of IFN-γ and IL-6 (Figure 3). The effect of DS-SILY_20_ on the production of the four pro-inflammatory cytokines was evaluated in both proliferative and quiescent SMC cultures. For proliferative SMC cultures, a general trend was observed such that as the concentration of DS-SILY_20_ increased, cytokine production decreased. Significant reductions in IFN-γ, IL-6, and TNF-α expression were exhibited with 10 μM DS-SILY_20_ treatment, while a significant decrease in IL-1β production was achieved using both 1 and 10 μM DS-SILY_20_. A similar trend was observed in quiescent SMC cultures with respect to IFN-γ production, where expression of the cytokine decreased as the concentration of DS-SILY_20_ increased; IFN-γin cultures treated with 10 μM DS-SILY_20_ decreased by ~41% compared to controls. The addition of 0.1, 1, and 10 μM DS-SILY_20_ to quiescent SMC cultures also resulted in significantly decreased TNF-αexpression. Interestingly, IL-1β and IL-6 production in cultures treated with 1 or 10 μM DS-SILY_20_ were not different from controls. However, an increase in IL-1β and IL-6 production occurred in quiescent cultures that were exposed to low concentrations of DS-SILY_20_.

### Thrombomodulin Expression

**Figure 4:**
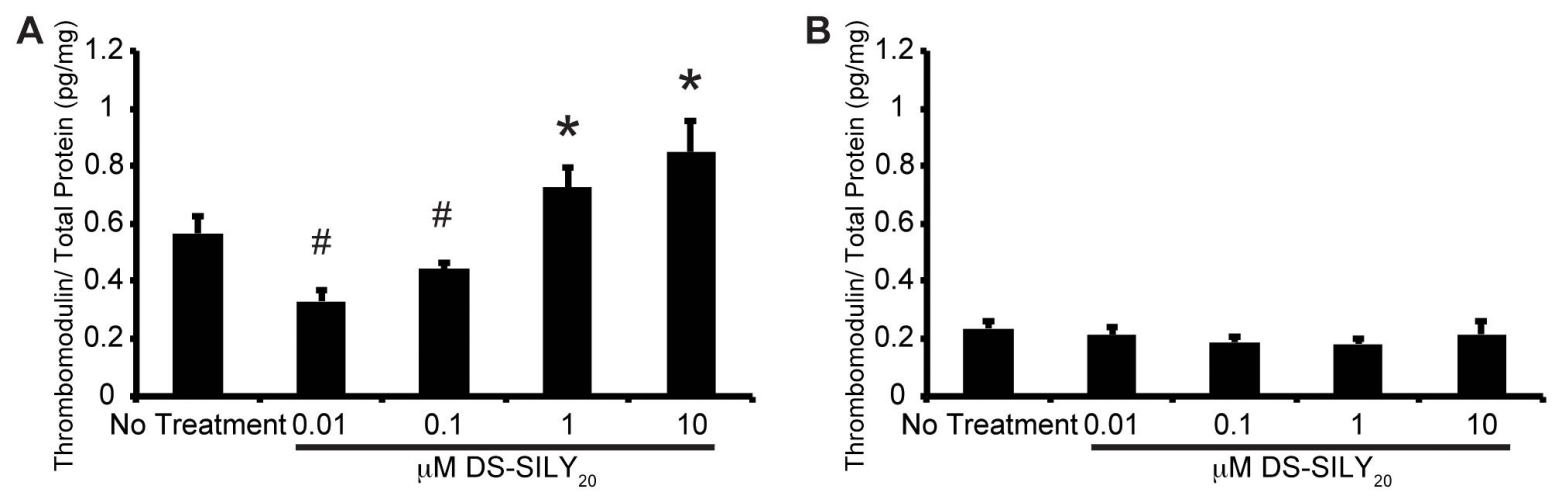
Thrombomodulin Production. Thrombomodulin expression from (A) proliferative and (B) quiescent SMCs in response to treatment with DS-SILY_20_. Thrombomodulin expression decreased with the addition low concentrations of DS-SILY_20_; however, the addition of either 1 or 10 μM DS-SILY_20_ resulted in significantly increased thrombomodulin production. Thrombomodulin production was not altered with the addition of DS-SILY_20_ in quiescent SMC cultures. * represents significant increase from control non-treated cells; # represents significant decrease from control non-treated cells. (N>4).

Thrombomodulin produced by SMCs in culture was analyzed via MSD Sector Imager. Control non-treated proliferative SMCs cultures exhibited significantly increased amounts of thrombomodulin compared to quiescent SMC cultures (Figure 4). The addition of 0.01 μM DS-SILY_20_ to proliferative cultures was found to decrease thrombomodulin expression by approximately 60% compared to control cultures. However, as the concentration of DS-SILY_20_ increased, thrombomodulin expression in proliferative SMCs also increased. At the highest concentrations of DS-SILY_20_ tested, thrombomodulin production was approximately 25% greater compared to non-treated proliferative cultures. Unlike proliferative SMCs, the addition of DS-SILY_20_ at any concentration did not alter thrombomodulin production in quiescent SMCs. 

### Analysis of Platelet Deposition and Activation on Balloon Injured Arteries

**Figure 5:**
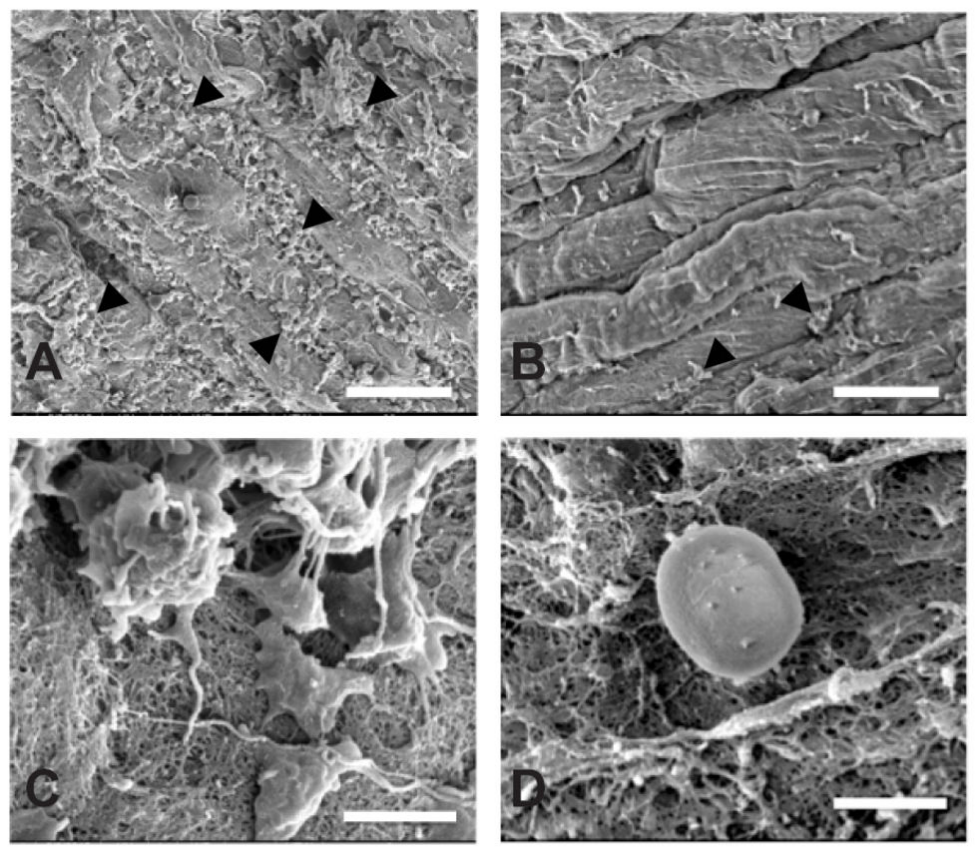
Platelet Deposition and Activation. SEM images of acute platelet response on artery wall in vessels treated with (A, C) DS-SILY_20_ and (B, D) sham saline control. Saline-treated arteries exhibited significant platelet coverage with numerous projections extending from activated platelets; platelets were scarcely visible on denuded arteries treated with DS-SILY_20_. Arrowheads indicate areas of platelet coverage. Magnification = 3,500x (A, B); 35,000x (C, D). Scale bar = 20 μm (A, B); 2 μm (C, D).

Three Ossabaw miniature pigs underwent angioplasty procedures and DS-SILY_20_ was delivered locally to endothelium-denuded arteries through a porous PTFE balloon catheter. To assess the acute response to balloon injury, platelet deposition on arteries was visualized via scanning electron microscopy. Platelets were scarcely visible on denuded arteries treated with DS-SILY_20_, compared to significant platelet coverage of the artery wall in saline-treated arteries (Figure 5A & B). At high magnification of the vessel walls, platelets attached on saline-treated arteries show numerous projections and spreading of platelets in addition to nascent fibrin strands, indicating platelet activation and early thrombus formation (Figure 5C). However, the few platelets seen on DS-SILY_20_ treated artery walls maintain a largely rounded morphology signifying inhibition of platelet activation (Figure 5D). Additional evidence for effective balloon denudation of the endothelium was the completely abolished endothelium-dependent relaxation to bradykinin (data not shown). 

### Analysis of Neointimal Hyperplasia at 1-Month Recovery

**Figure 6:**
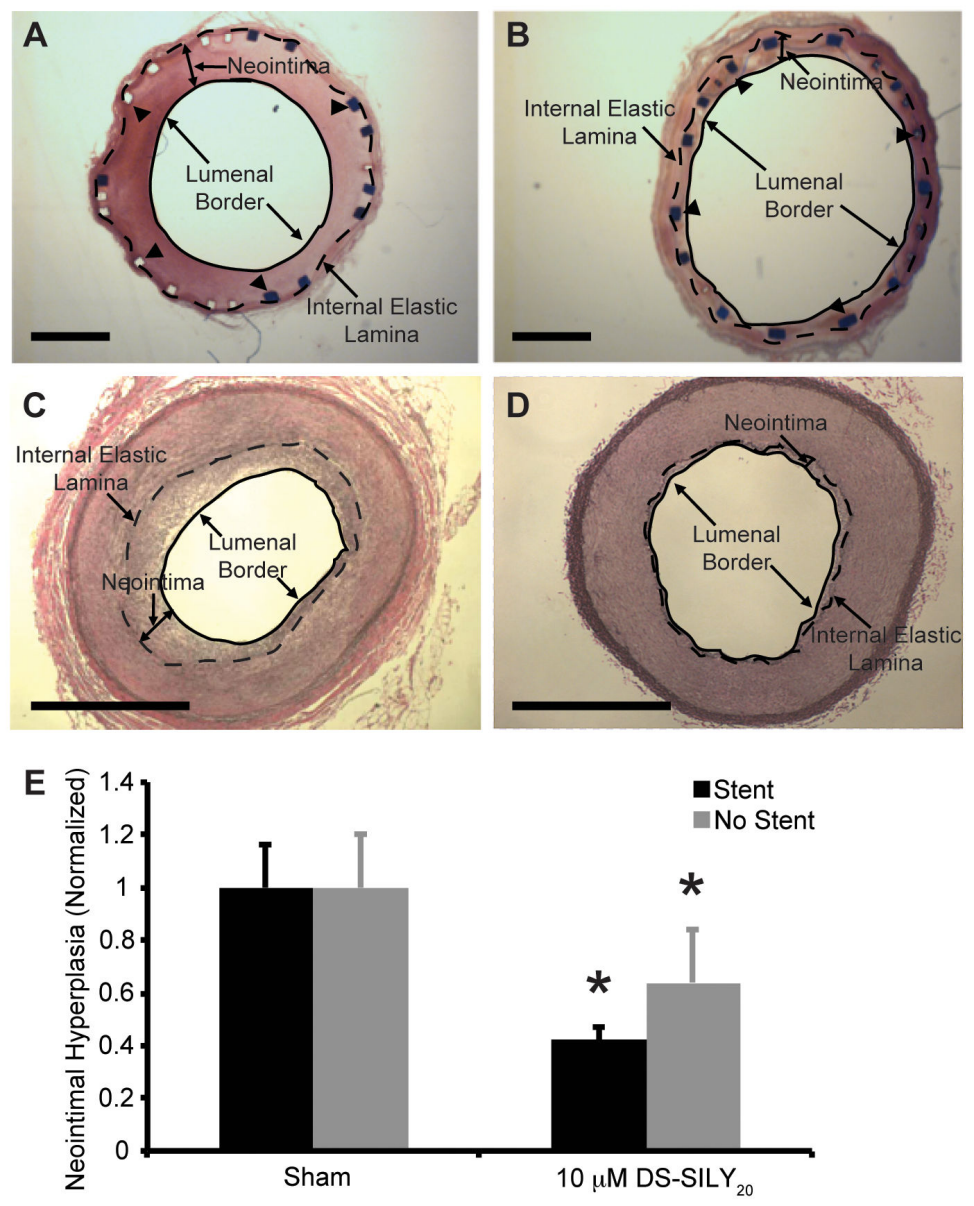
Neointimal Hyperplasia. Representative histology sections of (A, B) arteries with stents and (C, D) arteries without stents treated with (A, C) saline or (B, D) 10 μM DS-SILY_20_. Arrowheads indicate location of some stent struts; internal elastic lamina (dotted line) and lumenal border (solid line) are identified, indicating the boundaries of the neointima formed following injury. (E) Neointimal hyperplasia was quantified by measuring the distance from a stent strut or the elastic lamina to the vessel lumen in arteries with or without stents, respectively. A significant reduction in neointimal hyperplasia with DS-SILY_20_ treatment was observed, compared with saline sham controls, for both stented and non-stented artery segments. Six measurements were taken for each artery. Analysis with stents: sham (n=4), DS-SILY_20_ (n=3); without stents: sham (n=8), DS-SILY_20_ (n=5). Scale bar = 1 mm.* represents significance from sham-treated vessels.

Neointimal hyperplasia was assessed by histology after 1-month recovery in endothelium-denuded arteries treated with 10 μM DS-SILY_20_ or saline. Neointimal hyperplasia is observed with and without stents in sham controls, whereas minimal hyperplasia is observed in DS-SILY treated arteries (Figure 6A-D). A significant reduction in neointimal hyperplasia with DS-SILY_20_ treatment was observed, compared with saline sham controls, for both stented and non-stented artery segments (Figure 6E).

## Discussion

A detrimental consequence following PCI is injury to the vessel wall during balloon expansion, which triggers an array of mechanical and biological processes, leading to the occurrence of thrombosis, neointimal hyperplasia, and restenosis[37]. While many different cell types and processes are involved in the healing response of the injured vessel, previous studies have shown that SMC activation and extracellular matrix deposition play important roles in intimal hyperplasia after balloon injury[11-13]. We demonstrate here the use of an antithrombotic biomolecule, termed DS-SILY, to control SMC proliferation, migration, protein synthesis, cytokine excretion, and vascular injury marker production of both proliferative and quiescent SMCs *in vitro*. No measurable changes in metabolic activity were observed in either proliferative or quiescent SMC cultures following treatment with DS-SILY_20_. Furthermore, we examined the effects of this molecule on platelet adhesion and activation *in vivo*, as well as on intimal hyperplasia using an Ossabaw pig model.

Following balloon injury, SMCs become active, proliferating and migrating into the intima of the blood vessel wall, as well as secreting ECM proteins and cytokines. Thus, to best characterize the effects of our therapeutic, DS-SILY, we examined its efficacy on both proliferative and quiescent SMC cultures. Proliferative SMCs demonstrated increased proliferation and motility compared to quiescent SMCs. The addition of DS-SILY_20_ to cultures caused a dose dependent decrease of SMC proliferation for proliferative cultures. Previous reports examining SMC behavior in response to decorin treatment indicated decreased DNA synthesis in SMCs *in vitro* with the addition of decorin, similar to the results exhibited in this study[23]. While DS-SILY_20_ elicited a measurable response in proliferative cultures, no change in proliferation was observed in quiescent cultures treated with any concentration of DS-SILY_20_. This result is not unexpected as quiescent SMCs are already in a low proliferative state.

In this study, a dose-dependent decrease of SMC migration was observed for both proliferative and quiescent SMC cultures, where at 10 μM DS-SILY_20_ SMC migration was reduced by 95% and 47% for proliferative and quiescent cultures, respectively, compared to controls. Previous investigations of SMC migration in response to decorin treatment indicate a similar phenomenon, where the addition of decorin to *in vitro* cultures decreased SMC migration compared to non-treated controls[23]. While decreased migration in this previous work could be attributed to either the protein core or the DS glycosaminoglycan side chain, or a synergistic effect of both components, we demonstrate here that the DS glycosaminoglycan plays a significant role in determining the mobility of SMCs. This phenomenon has also been reported previously, where SMCs cultured with exogenous DS exhibited a 25% reduction in SMC migration[38]. While it is not known exactly why DS-SILY_20_ exhibited a larger anti-migratory effect compared to previous work with exogenous DS, it is speculated that the difference in the physical presentation of the molecules to cells, exogenous compared to collagen-bound, may play a role.

SMC migration into the intimal layer of the vessel following vessel injury is generally accompanied by increased protein synthesis from the activated cells[39]. Thus, as activated SMCs express increased protein compared to SMCs found in healthy vessel walls, the ability to control protein synthesis is also important. As expected, protein synthesis, as detected by fluorescently labeling newly synthesized proteins, was significantly increased in proliferative SMC cultures compared to quiescent cells. This is consistent with the finding that proliferative cultures contained ~60% more protein per cell than quiescent cultures. The addition of DS-SILY_20_ to cultures did not alter protein synthesis occurring in quiescent SMCs compared to controls; this indicates that the therapeutic likely does not disrupt important cellular processes associated with quiescent cell function. When applied to proliferative cultures, a significant decrease in protein expression was achieved with 10 μM DS-SILY_20_, compared to non-treatment controls. The ability of DS-SILY to suppress protein synthesis in proliferative smooth muscle cells may contribute to less protein deposition in the vascular wall when DS-SILY is applied to the vessel following balloon angioplasty. Interestingly, SMCs treated with decorin have demonstrated similar results, where SMC collagen synthesis decreased with the addition of decorin to cultures[23]. In addition, treatment with DS alone has been shown to decrease collagen production in other types of tissues, including obstructed kidney tissue[40].

While treatment with 10 μM DS-SILY_20_ significantly reduced protein synthesis compared to non-treated proliferative cultures, the reduction in protein synthesis remained significantly increased compared to levels exhibited by quiescent SMC cultures. Furthermore, no significant changes in the amount of protein synthesized from proliferative cultures treated with lower concentrations of DS-SILY_20_ was observed compared to non-treated controls. Taken together, these results indicate that increased concentrations of DS-SILY_20_ may need to be utilized in order to decrease the amount of protein produced by proliferative SMCs to levels similar to that of quiescent cells. Further exploration into this phenomenon will need to be conducted.

Following vessel injury, stimulated SMCs actively participate in the inflammatory cycle by producing and secreting a range of pro-inflammatory factors[41]. It has previously been demonstrated that cytokine secreting cells are more likely to be actively proliferating cells involved in intimal hyperplasia[41]. In this study, proliferative SMCs expressed more TNF-α and IL-1β than quiescent cells; however, both IFN-γ and IL-6 remained similar between cultures. Thus, findings from this study indicate that the transition between SMC phenotypes only affects the production of certain cytokines, while the expression of others remains unaltered. As such, reducing the expression of the inflammatory cytokines directly involved in the onset of intimal hyperplasia, in part by decreasing the participation of SMCs in the inflammatory progression that occurs following PCI, may help minimize the restenotic cascade[42].

One of the key findings in this study is the ability of DS-SILY_20_ to reduce the production of pro-inflammatory cytokines elicited from SMCs. The addition of 10 μM DS-SILY_20_ to proliferative cultures decreased the production of all four inflammatory cytokines investigated in this work. In quiescent cultures, the addition of high concentrations of DS-SILY_20_ also resulted in the reduction of both IFN-γ and TNF-α. Interestingly, the treatment of quiescent cultures with low concentrations of DS-SILY_20_ caused an increase of IL-1β and IL-6. This observed increase in IL-1β and IL-6 corresponds with a slight increase in quiescent SMC migration that was also observed in this work, indicating that low concentrations of the compound stimulates a small level of smooth muscle cell repair and remodeling[43].

In addition to the effect of DS-SILY_20_ on SMC cytokine production, the antithrombotic therapeutic influences cellular expression of thrombomodulin, a transmembrane glycoprotein, which plays an important role in maintaining vascular thromboresistance. During the coagulation cascade, thrombomodulin forms a complex with thrombin, allowing for the activation of protein C and thus, indirectly increasing fibrinolysis and inhibiting blood coagulation[44]. Similar to previous reports, thrombomodulin levels were almost indiscernible in quiescent SMC cultures[45]. A decrease in thrombomodulin production was exhibited in proliferative SMC cultures with low concentrations of DS-SILY. While this response is not ideal, the concerns associated with decreased thrombomodulin expression may be overshadowed by the ability of DS-SILY_20_ to reduce other attributes associated with proliferative SMCs, as demonstrated in both the *in vitro* and *in vivo* studies within this work. However, we also demonstrate that the expression of thrombomodulin increases in proliferative SMCs treated with 1 or 10 μM DS-SILY_20_. As it has previously been determined that the overexpression of thrombomodulin, or similarly, the systemic administration of thrombomodulin, reduces inflammatory cell infiltration and neointimal formation in several animal models[46,47], the ability of DS-SILY_20_ to upregulate thrombomodulin in proliferative, unhealthy SMCs may serve as another important mechanism in the prevention of restenosis. This suggests that higher treatment concentrations of DS-SILY (~10 μM) are warranted. 

An *in vivo* proof-of-principle was evaluated in Ossabaw miniature pigs in an effort to further assess the ability of DS-SILY_20_ to inhibit intimal hyperplasia. Previously, we have demonstrated the ability of DS-SILY to specifically bind to type-I collagen, serving as a barrier to platelet adhesion and activation *in vitro*[30]. Here we further examined the ability of DS-SILY_20_ to block platelet adhesion and activation *in vivo* using an Ossabaw miniature pig model. Following balloon angioplasty-induced denudation of the endothelium, solutions of 10 μM DS-SILY_20_ or the sham control were delivered to the injured arterial wall via porous balloon catheter. Short-term effects on platelet activation were examined. Examination of denuded artery walls indicated that platelets were scarcely visible on DS-SILY_20_ treated vessels, while significant platelet coverage was visualized on saline-treated arteries. More interestingly, platelets attached to saline-treated arteries show numerous projections and spreading of platelets, which is indicative of platelet activation[48]. However, platelets on DS-SILY_20_ treated artery walls maintain a largely rounded morphology signifying inhibition of platelet activation. These findings demonstrate that *in vivo* delivery of DS-SILY_20_ is achievable and indicate efficacy of inhibited platelet activation on the endothelium-denuded artery wall.

Furthermore, to examine the effects of DS-SILY_20_ on reducing neointimal hyperplasia, artery histology was assessed after a 1-month recovery following balloon angioplasty in stented and non-stented arteries. Intimal hyperplasia was significantly reduced in vessels treated with DS-SILY_20_ compared to sham controls. Based on the ability of DS-SILY_20_ to inhibit SMC proliferation, migration, and protein synthesis *in vitro*, it is possible that DS-SILY_20_ prevents intimal hyperplasia through multiple mechanisms culminating in the direct suppression of the cellular behavior that is stimulated by direct mechanical injury of the blood vessel during balloon expansion. As DS-SILY also binds to the luminal surface of the injured artery and inhibits platelet binding and activation, it is also likely that DS-SILY indirectly prevents hyperplasia through an antithrombotic mode of action, as shown in our previous work[30]. Interestingly, a significant reduction in intimal hyperplasia was observed in both stented and non-stented vessels, indicating the delivery of DS-SILY_20_ from balloon catheters alone, without additional stent implantation, may alone provide medically significant vessel healing. 

In conclusion, DS-SILY demonstrates multiple biological activities that may all synergistically contribute to an improved treatment paradigm for balloon angioplasty. DS-SILY_20_ directly inhibited platelet binding and activation on denuded arteries *in vivo*. In addition, *in vitro* the therapeutic was shown to stimulate thrombomodulin production in proliferative SMCs, thus potentially contributing to the antithrombotic potential of DS-SILY *in vivo*. The decorin mimic also suppressed SMC proliferation, migration, and protein synthesis in proliferative SMC cultures *in vitro*, which may contribute to the inhibition of intimal hyperplasia seen following DS-SILY treatment after balloon angioplasty *in vivo*. Finally, DS-SILY_20_ suppressed SMC pro-inflammatory cytokine production *in vitro*, which may also prove critical in its ability to inhibit intimal hyperplasia *in vivo*. Future studies are aimed at better elucidating the mechanisms through which these multiple activities contribute to improved vessel healing following PCI *in vivo*. 

## Supporting Information

Figure S1 **Schematic Representation of Fluorescently Labeled Protein.** (A) Following incubation with DS-SILY_20_, cultures were doped with L-azidohomoalanine (AHA) in serum-free media for 4 hrs. Cells were then rinsed in PBS and incubated in co-culture media overnight to allow protein production. (B) Cultures were fixed and permeabilized, prior to the selective conjugation of Alexa Fluor 594 (AF-594) via copper-catalyzed azide-alkyne ligation. *De* *novo* protein synthesis was then visualized by detecting fluorescent tag attached directly to the unnatural amino acid.
(TIF)
