# Supplementary figures and images for: Decorin Mimic Inhibits Vascular Smooth Muscle Proliferation and Migration
Source: PLoS One. 2013 Nov 22;8(11):e82456. doi: 10.1371/journal.pone.0082456 (PMC3838406; doi:10.1371/journal.pone.0082456)

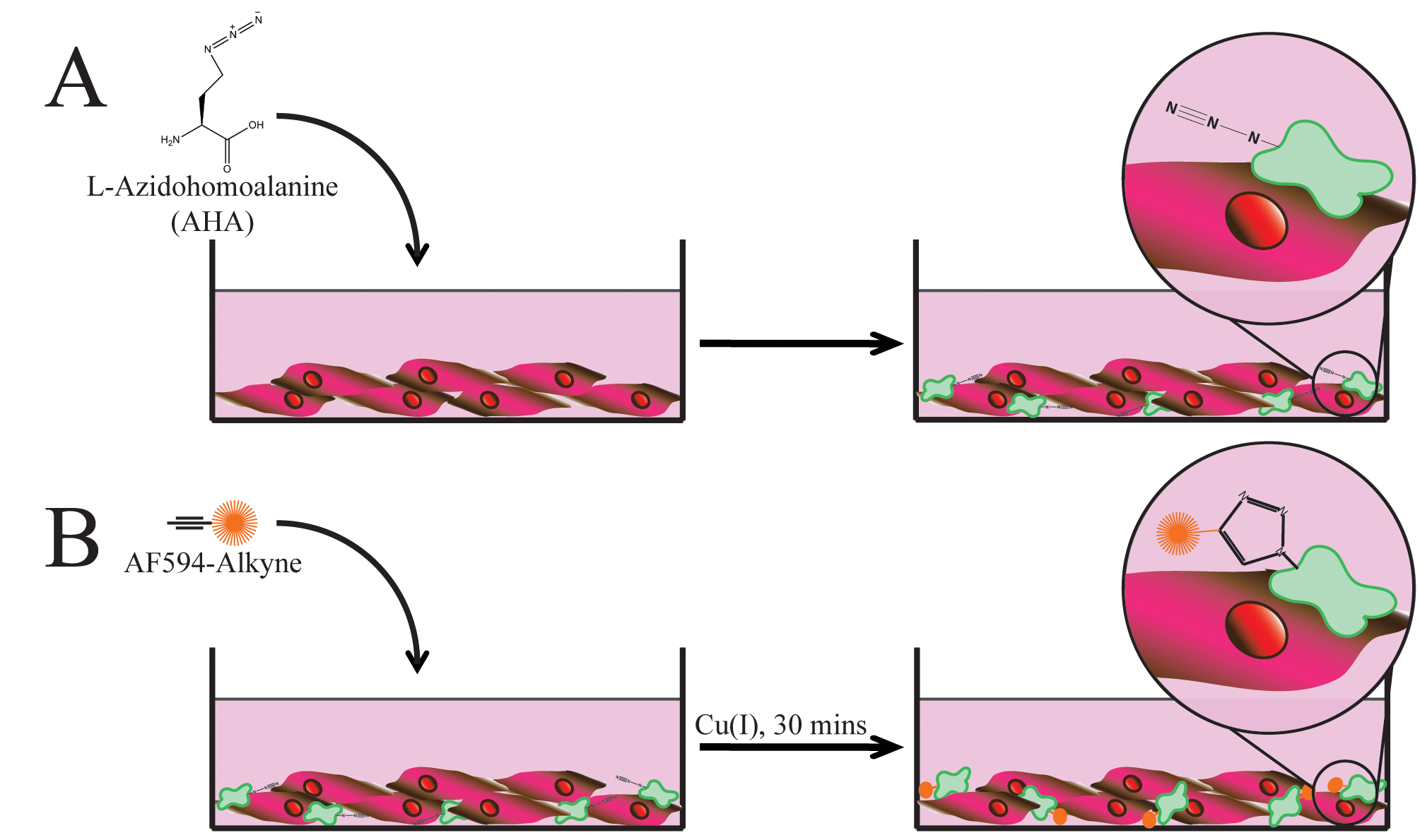

Supplement: Figure S1 — Schematic Representation of Fluorescently Labeled Protein. (A) Following incubation with DS-SILY20, cultures were doped with L-azidohomoalanine (AHA) in serum-free media for 4 hrs. Cells were then rinsed in PBS and incubated in co-culture media overnight to allow protein production. (B) Cultures were fixed and permeabilized, prior to the selective conjugation of Alexa Fluor 594 (AF-594) via copper-catalyzed azide-alkyne ligation. De novo protein synthesis was then visualized by detecting fluorescent tag attached directly to the unnatural amino acid. (TIF) [file pone.0082456.s001.tif]
